# Resveratrol Improves the Progression of Osteoarthritis by Regulating the SIRT1-FoxO1 Pathway-Mediated Cholesterol Metabolism

**DOI:** 10.1155/2023/2936236

**Published:** 2023-01-04

**Authors:** ChuanCai Liang, Hengte Xing, ChenYu Wang, XiongFeng Xu, Yarong Hao, Bo Qiu

**Affiliations:** ^1^Department of Orthopedics, Renmin Hospital of Wuhan University, Wuhan, China; ^2^Department of Geriatrics, Renmin Hospital of Wuhan University, Wuhan, China

## Abstract

Osteoarthritis (OA) is considered a metabolic disorder. This study investigated the effect of resveratrol (RES) on cholesterol accumulation in osteoarthritic articular cartilage via the silent information regulator 1 (SIRT1)/forkhead transcription factor (FoxO1) pathway. Interleukin (IL)-1*β*-treated chondrocytes that mimic OA chondrocytes were used in *in vitro* experiments. The optimal RES concentration was selected based on the results of chondrocyte proliferation in the Cell Counting Kit-8 assay. Western blotting, immunofluorescence, and reverse transcription-quantitative polymerase chain reaction were performed. For the animal experiments, mice were randomly divided into the RES group (*n* = 15), medial meniscus destabilization group (*n* = 15), and sham group (*n* = 15), and each group received the same dose of RES or saline. Articular cartilage tissue was obtained eight weeks after surgery for relevant histological analysis. Clinical tissue test results suggest that downregulation of the SIRT1/FoxO1 pathway is associated with cholesterol buildup in OA chondrocytes. For the *in vitro* studies, RES increased the expression of SIRT1 and phosphorylation of FoxO1 in IL-1*β*-treated chondrocytes, promoted the expression of cholesterol efflux factor liver X receptor alpha (LXR*α*), and inhibited the expression of cholesterol synthesis-associated factor sterol-regulatory element binding proteins 2 (SREBP2). This reduced IL-1*β*-induced chondrocytes cholesterol accumulation. SIRT1 inhibition prevented the RES-mediated reduction in cholesterol buildup. Inhibiting *FoxO1* but not *SIRT1* reduced *FoxO1* phosphorylation and increased cholesterol buildup in cultured chondrocytes. Additionally, *in vivo* experiments have shown that RES can alleviate cholesterol buildup and pathological changes in OA cartilage. Our findings suggest that RES regulates cholesterol buildup in osteoarthritic articular cartilage via the SIRT1/FoxO1 pathway, thereby improving the progression of OA.

## 1. Introduction

Osteoarthritis (OA) is an age-related condition of the articular cartilage that can cause chronic pain and limit joint function. Current treatments do not reverse the changes in OA but only reduce joint pain [1]. Historically, OA has been considered a degenerative joint disease largely associated with sex, prior injuries, and age [2]. It is related to metabolic disorders that speed up the progression of OA pathology [3]. This strengthens the association between OA and metabolic syndrome [4]. Cholesterol buildup in chondrocytes plays a significant role in the pathogenesis of OA and induces the chondrocyte OA phenotype [5, 6]. Recent studies have shown the presence of specific lipid deposits in OA chondrocytes. There was a marked increase in cholesterol synthesis and a decrease in efflux, leading to an accumulation of intracellular cholesterol [7]. In addition, Ali et al. found that cholesterol accumulation in chondrocytes of OA patients inhibited the synthesis of extracellular substances and reduced the quality of articular cartilage [8]. Therefore, regulating cholesterol metabolism, particularly in articular cartilage, might be a potential target for treating OA.

Resveratrol (RES) is a natural antioxidant [9]. There is growing evidence that RES plays an important role in orthopedic, cardiovascular, and oncological diseases. The main reason is that RES has lipid-regulating, anti-inflammatory, antioxidant, and antiageing activities [9–11]. Animal studies have shown that resveratrol inhibits chondrocyte autophagy, apoptosis, and extracellular matrix degradation and attenuates the progression of OA [12]. Another study showed that resveratrol protects articular cartilage by activating the silent information regulator (SIRT1)/forkhead transcription factor (FoxO1) pathway, promoting chondrocyte autophagy and inhibiting apoptosis, thereby inhibiting extracellular matrix degradation [13]. Although many mechanisms may mediate the role of resveratrol in the prevention and treatment of OA, most studies have focused on its anti-inflammatory, chondrogenic matrix-protective, or antiaging effects. Therefore, the potential effects or mechanisms of RES on the development of OA remain underexplored, and further studies are needed to explore the relevant signaling pathways.

Silent information regulators are essential in gene sequencing, genome instability, and cell lifespan. FoxO1 expression is highest in chondrocytes and significantly regulates growth, senescence, and inflammatory stress [14]. The *FoxO1* binding site lies in the promoter region of the cholesterol synthesis gene sterol-regulatory element binding proteins 2 (SREBP2), which promotes the phosphorylation of *FoxO1* and upregulates *SREBP-2* gene expression [15]. FoxO1 expression was reduced in the hepatocytes of hyperlipidemic rats, which improved *SREBP-2* gene expression and increased cholesterol synthesis. In contrast, after *SIRT1* was activated, *FoxO1* expression increased, *SREBP-2* expression decreased, and hepatocyte cholesterol synthesis was inhibited [16]. The inhibition of HCV replication significantly increased the expression of *SIRT1* and enhanced *FoxO1* activity in Huh-7.5 cells carrying HCV replicons, downregulating the transcription of *SREBP-2* and other genes and lowering lipid synthesis [15]. However, the relationship between cholesterol buildup in OA cartilage and the SIRT1/FoxO1 pathway has not been reported yet [17]. Therefore, targeted modulation of the SIRT1/FoxO1 pathway may be a potential approach to regulate cholesterol accumulation in OA cartilage.

Therefore, the aim of this study was to investigate the mitigating effects and mechanisms of resveratrol on lipid accumulation in OA chondrocytes via the SIRT1/FoxO1 signaling pathway.

## 2. Materials and Methods

### 2.1. Cell Counting Kit-8 (CCK-8) Assay for Cell Activity

Influence of RES on chondrocyte viability: normal chondrocytes and IL-1*β*-induced chondrocytes were placed in 96-well plates overnight. They were treated with various RES concentrations and incubated at 37°C for 24 h. The cell viability was then determined after the CCK-8 solution was added to the cell culture wells, and the incubation was continued for 4 h. The test was performed in triplicate.

### 2.2. Chondrocyte Culture

Mice (Shulb, Wuhan, China) were sacrificed, and bilateral knee cartilage tissue was extracted. The cartilage tissue was cut with scissors in a sterile laboratory environment, after which it was soaked in 0.1% trypsin (Beyotime, Shanghai, China) for 1 h and cultured in a cell culture chamber overnight. The digested cells were collected and resuspended in a DMEM/F-12 medium (BI, Kibbutz Beit Haemek, Israel). The chondrocytes were treated with the *SIRT1* inhibitor EX-527 (10 *μ*M/mL), the *FoxO1* inhibitor AS (1 *μ*M/mL), and resveratrol (50 *μ*M) for 6 h to inhibit the SIRT1/FoxO1 signaling pathway.

### 2.3. Human Cartilage Tissue

Comparison of cholesterol accumulation in OA chondrocytes and SIRT1-FoxO1 pathway validation based on clinical specimens: three male and three female patients with OA or femoral neck fractures (mean age 70.59 ± 2.57 years) who underwent arthroplasty for cartilage tissue were used as the OA and control groups, respectively. The cartilage tissue was fixed in 4% paraformaldehyde and embedded and sectioned in paraffin, and each section was stained with hematoxylin and eosin (H&E) to assess the degree of cartilage destruction. Articular cartilage tissue was cut into small pieces and digested, and the obtained chondrocytes were cultured. The first generation of cultured chondrocytes was used in all the experiments. The Ethics Committee approved the study at Wuhan University, and each participant provided informed consent.

### 2.4. RT-qPCR

The expression levels of *COL-II*, *ADAMTS5*, *MMP13*, *SOX6*, *SIRT1*, *FoxO1*, *ABCA1*, *ApoA1*, *GAPDH*, *SREBP-2*, and *HMGCR* were analyzed. Total cellular RNA was extracted using a TRIzol reagent (Ambion, China). cDNA was synthesized using a cDNA synthesis kit (VAZYME, Nanjing, China). The target genes were multiplied using the SYBR Premix Ex Taq kit (VAZYME, Nanjing, China). The mRNA levels were normalized to endogenous GAPDH, and the target gene expression levels were analyzed by the 2^−ΔΔCT^ method. The primers used are provided in Table 1.

### 2.5. Western Blotting

Western blot analysis was used to analyze the protein expression levels of *COL-II*, *ADAMTS5*, *SOX6*, *MMP13*, *SIRT1*, *FoxO1*, *p-FoxO1*, *ABCA1*, *ApoA1*, *LXRα*, *SREBP-2*, and *HMGCR* in the chondrocytes. The cells were cultured for 24 h, after which the chondrocytes were lysed using cell lysate to obtain proteins; the target protein content was analyzed using a bicinchoninic acid assay kit (Bain Marie, China). The collected proteins were stored at -20°C. The target proteins were electrophoresed separately, transferred, immersed in a warm blocking buffer for 1 h, incubated with a primary antibody for 6 h, and incubated with a secondary antibody (horseradish peroxidase-labeled goat antirabbit, Wuhan Bost Biotech Co., Ltd.) (1 : 50,000) for 1 h. The strip was developed after the final wash. GAPDH was used as the standard endogenous protein. ImageJ was used to convert the image to grayscale. The test was performed in triplicate. The antibodies used are provided in Table 2.

### 2.6. Immunofluorescence Staining

The chondrocytes were first fixed in 4% paraformaldehyde for 20 min and then permeabilized with phosphate-buffered saline with Tween for 15 min. Diluted target protein *p-FoxO1* primary antibody (Affinity, AF3416) (1 : 1000) was added drop by drop, and the cells were incubated at 4°C overnight. Goat anti-rabbit immunoglobulin (Ig)G antibody (Affinity, AF0135) (1 : 5000) was added drop by drop, and the cells were incubated at 37°C for 1 h. Further, a DAPI solution was added, and an inverted fluorescence microscope was used to capture the images. The test was performed in triplicate.

### 2.7. Measurement of Cellular Free and Total Cholesterol Levels

Kits for detecting free and total cholesterol in cells were purchased from Solarbio Bioscience and Technology (Shanghai, China) and Applygen Ltd. (Beijing, China). The specific procedures were carried out following the instructions provided with the kits.

### 2.8. Animal Models

Sixty 4-week-old mice were obtained from Rat Leprechaun, China. The mice in this study had free access to normal food and water prior to any experimental procedure. The experimental animal procedures adhered to the National Institutes of Health guidelines for the use of laboratory animals and were approved by the Animal Use Committee of the Renmin Hospital of Wuhan University. Mice underwent surgery for medial meniscal instability (DMM), producing an animal OA model. The mice were anesthetized by injecting 30 mg/kg of 2% (w/v) pentobarbital. The medial meniscus was then removed after dissecting the medial capsule of the right knee. Incisions were made in the medial meniscus and tibial ligament. Microsurgical scissors were used. A sham operation, consisting of an arthrotomy without cutting the medial meniscus ligament, was performed simultaneously to serve as a control group. Mice were randomly divided into three groups. Sham group (*n* = 15), DMM group (*n* = 15), and RES+DMM group (*n* = 15). Mice in the DMM and sham groups were injected with normal saline immediately after surgery, while mice in the RES+DMM group were intraperitoneally injected RES (50 mg/kg/day) in saline. Mice were killed at the end of week eight using an overdose of sodium pentobarbital.

### 2.9. Histological Evaluation

Cartilage tissue was fixed with 4% paraformaldehyde and decalcified with 10% ethylenediamine tetraacetic acid solution for a month before histological analysis. Decalcified tissue was embedded in paraffin, the paraffin was sectioned, and the sections were stained with H&E and saffron O. The cellular morphology of cartilage and subchondral bone was assessed using the Osteoarthritis Research Society International (OARSI) scoring system [18].

### 2.10. Reagents

Resveratrol (RES, No. R107315, 99% purity), Selisistat (EX-527, No. S1541, 99% concentration), and AS1842856 (AS, No. 88222, 99% concentration) were obtained from MacLean Biotech (Shanghai, China).

### 2.11. Statistical Analysis

Each test was repeated three or more times. Data are presented as mean ± standard error of the mean. The data were processed with GraphPad Prism 8.0. One-way ANOVA analysis of factors was used to analyze differences between multiple groups, and a *p* value < 0.05 was considered statistically significant.

## 3. Results

### 3.1. Influence of RES on Chondrocyte Viability

**Figure 1:**
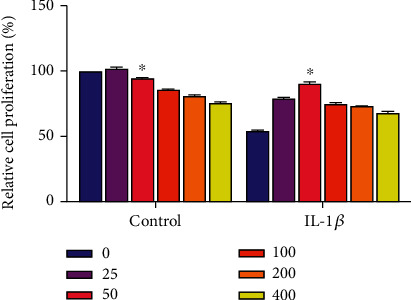
Effect of resveratrol on chondrocyte proliferation determined by the Cell Counting Kit-8 assay to select the optimal drug concentration for subsequent *in vitro* experiments.

We tested the effects of RES on normal chondrocytes and IL-1*β*-induced chondrotoxicity using the CCK-8 kit. Cells were treated with different concentrations of RES for 24 hours. The maximum RES concentration that was not cytotoxic, i.e., cell survival >90%, was selected for subsequent experiments, and the most suitable RES concentration was 50 *u*M based on the experimental results (Figure 1).

### 3.2. Comparison of Cholesterol Accumulation in OA Chondrocytes and SIRT1-FoxO1 Pathway Validation Based on Clinical Specimens

**Figure 2:**
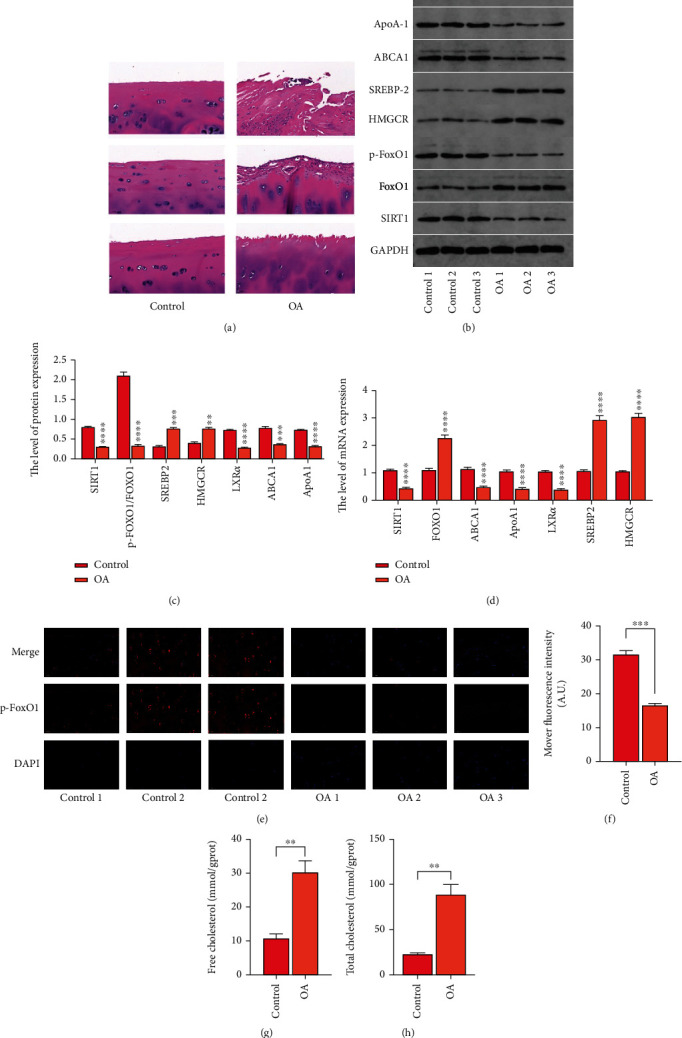
Correlation between cholesterol buildup in osteoarthritis (OA) chondrocytes and the SIRT1/FoxO1 pathway based on validation of clinical specimens. (a) The extent of OA pathology was assessed by hematoxylin and eosin staining clinical OA cartilage tissue. (b, c) Protein expression of *SIRT1*, *p-FoxO1/FoxO1*, *HMGCR*, *SREBP2*, *ABCA1*, *APOA1*, and *LXRα* in cartilage tissues were detected. (d) Reverse transcription-quantitative polymerase chain reaction analysis of the expression of *SIRT1*, *p-FoxO1/FoxO1*, *HMGCR*, *SREBP2*, *ABCA1*, and *LXRα*. (e, f) Immunofluorescence detection of p-FoxO1 protein content (×200) and quantitative analysis using ImageJ software. (g, h) Free and total cholesterol levels in cartilage tissues were quantified using the respective cholesterol assay kits. The values represent the mean ± standard deviation of three independent experiments. ^∗^*p* < 0.05, ^∗∗^*p* < 0.01, ^∗∗∗^*p* < 0.001, and ^∗∗∗∗^*p* < 0.0001.

We collected cartilage tissues from three patients with OA and three with femoral neck fractures. After H&E staining, the fracture group's four-layer cartilage structure was visible, and the chondrocytes were arranged neatly. The cartilage surface was continuously damaged and significantly lost and eroded in the OA group, leading to the formation of cracks (Figure 2(a)). The p-FoxO1 protein expression was detected by immunofluorescence (Figures 2(e) and 2(f)). Chondrocyte *p-FoxO1* expression was significantly lower in the OA group than in the control group. We examined the expression levels of *SIRT1*, *FoxO1*, *p-FoxO1/FoxO1*, and cholesterol metabolism-related genes (*HMGCR*, *SREBP2*, *ABCA1*, *ApoA1*, and *LXRα*) (Figures 2(b)–2(d)). *SIRT1*, *FoxO1*, *p-FoxO1*, *ABCA1*, *ApoA1*, and *LXRα* expression levels were lower in the chondrocytes of the OA group. *HMGCR* and *SREBP2* had significantly higher expression levels. Further, total and free cholesterol kits were used to measure chondrocyte cholesterol levels. The OA group's chondrocytes had significantly higher cholesterol levels (Figures 2(g) and 2(h)). In conclusion, SIRT1/FoxO1 pathway may regulate the cholesterol metabolism of OA chondrocytes, and the accumulation of cartilage cholesterol is correlated with the occurrence of OA.

### 3.3. Resveratrol Improves the Phenotype of IL-1*β*-Induced Chondrocytes via the SIRT1-FoxO1 Pathway

**Figure 3:**
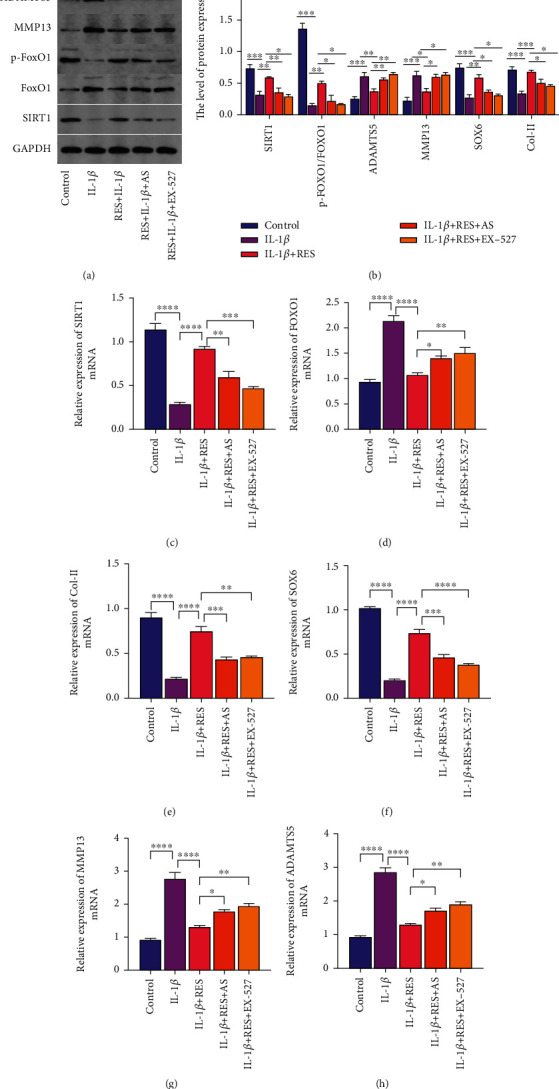
Resveratrol ameliorates the phenotype of interleukin-1*β*-induced chondrocytes via the SIRT1-FoxO1 pathway. (a, b) Western blot to detect the expression levels of the chondrocyte phenotype-related genes *MMP13*, *ADAMS5*, *COL-II*, and *SOX6*. (c–h) The mRNA levels of *SIRT1*, *p-FoxO1/FoxO1*, *MMP13*, *ADAMTS5*, *COL-II*, and *SOX6* were detected by reverse transcription-quantitative polymerase chain reaction. The values represent the mean ± standard deviation of three independent experiments. ^∗^*p* < 0.05, ^∗∗^*p* < 0.01, ^∗∗∗^*p* < 0.001, ^∗∗∗∗^*p* < 0.0001, and ns: not significant.

The expression levels of *SIRT1* and *p-FoxO1/FoxO1* were significantly reduced in IL-1*β*-induced chondrocytes, while the addition of RES significantly increased the expression levels (Figures 3(a)–3(d)). This suggests that RES activates the SIRT1/FoxO1 pathway.

We investigated the expression levels of *SOX6*, *COL-II*, *ADAMTS5*, and *MMP-13*, which are associated with chondrocyte phenotype, to explore further the role of the SIRT1-FoxO1 pathway on chondrocyte phenotype. IL-1*β* significantly reduced *SOX6* and *COL-II* levels while increasing *MMP13* and *ADAMTS5* levels (Figures 3(a), 3(b), and 3(e)–3(h)). The addition of RES significantly reduced the effect of IL-1*β* on chondrocytes (Figures 3(a), 3(b), and 3(e)–3(h)). The protective effect of RES on chondrocytes was significantly reduced when RES and EX-527 or AS were added (Figures 3(a), 3(b), and 3(e)–3(h)). Therefore, RES can effectively alleviate the IL-1*β*-induced chondrocytes phenotype by activating the SIRT1/FoxO1 pathway.

### 3.4. Resveratrol Inhibits IL-1*β*-Induced Chondrocytes Cholesterol Synthesis via the SIRT1-FoxO1 Pathway

**Figure 4:**
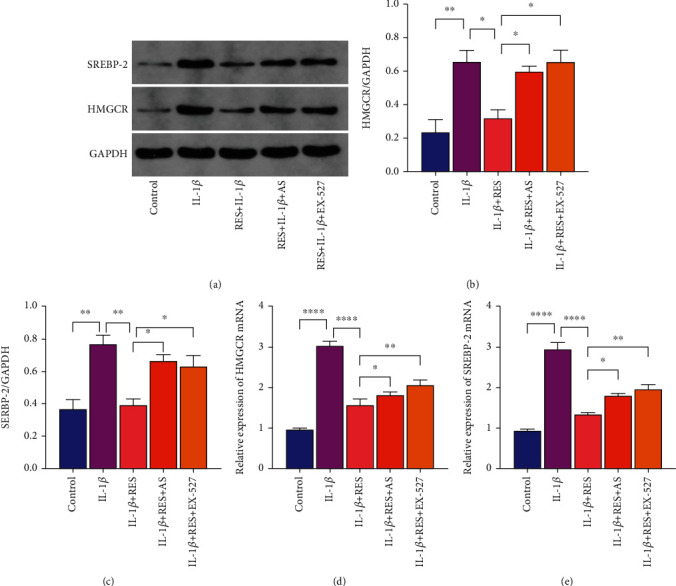
Resveratrol ameliorates IL-1*β*-induced chondrocytes cholesterol synthesis via the SIRT1-FoxO1 pathway. (a–c) Western blot analysis of protein expression levels of *SREBP-2* and *HMGCR*. (d, e) Levels of *SREBP-2* and *HMGCR* in each group of chondrocytes were quantified by qRT-PCR. The values represent the mean ± standard deviation of three independent experiments. ^∗^*p* < 0.05, ^∗∗^*p* < 0.01, ^∗∗∗^*p* < 0.001, and ^∗∗∗∗^*p* < 0.0001.

We investigated the effects of RES on cholesterol metabolism in chondrocytes to determine the mechanisms underlying the protective effects of RES on OA chondrocytes. IL-1*β* significantly promoted the expression of cholesterol synthesis-related factors *SREBP-2* and *HMGCR* (Figures 4(a)–4(g)). Following IL-1*β*-induced chondrocyte addition of RES, RES significantly reversed the effects of IL-1*β* on chondrocyte cholesterol synthesis (Figures 4(a)–4(e)). The reversal of IL-1-induced chondrocytes cholesterol synthesis was significantly reduced by the addition of RES and EX-527 or AS (Figures 4(a)–4(e)). Therefore, RES can effectively ameliorate IL-1*β*-induced chondrocytes cholesterol synthesis via the SIRT1-FoxO1 pathway.

### 3.5. Resveratrol Inhibits IL-1*β*-Induced Chondrocytes Cholesterol Efflux via the SIRT1-FoxO1 Pathway

**Figure 5:**
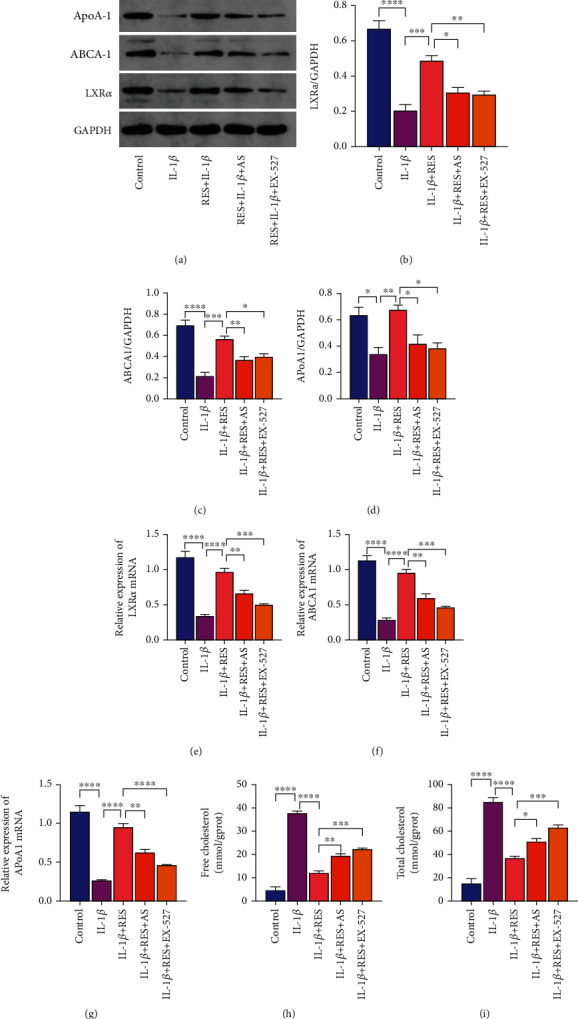
Resveratrol ameliorates IL-1*β*-induced chondrocytes cholesterol efflux via the SIRT1-FoxO1 pathway. (a–d) Western blot analysis of protein expression levels of *LXRα*, *ABCA1,* and *ApoA1*. (e–g) Levels of *ABCA1*, *ApoA1,* and *LXRα* in each group of chondrocytes were quantified by qRT-PCR. (h, i) Free and total cholesterol profiles of chondrocytes in each group were analyzed using the respective cholesterol assay kits. The values represent the mean ± standard deviation of three independent experiments. ^∗^*p* < 0.05, ^∗∗^*p* < 0.01, ^∗∗∗^*p* < 0.001, and ^∗∗∗∗^*p* < 0.0001.

IL-1*β* significantly inhibited the expression of cholesterol efflux-related factors *ABCA1*, *ApoA1,* and *LXRα* (Figures 5(a)–5(g)). RES significantly reversed the effects of IL-1*β* on chondrocyte cholesterol efflux following IL-1*β*-induced chondrocyte addition of RES (Figures 5(a)–5(g)). The reversal effect of RES on IL-1-induced chondrocytes cholesterol efflux was significantly reduced by the addition of RES and EX-527 or AS (Figures 5(a)–5(g)). The above results were also verified by measuring the total cellular and free cholesterol (Figures 5(h) and 5(i)) levels. Therefore, RES could reduce IL-1*β*-induced chondrocytes free and total cholesterol levels. In conclusion, RES can effectively ameliorate IL-1*β*-induced chondrocytes cholesterol accumulation via the SIRT1-FoxO1 pathway.

### 3.6. RES Ameliorates OA Development in DMM Mice Models

**Figure 6:**
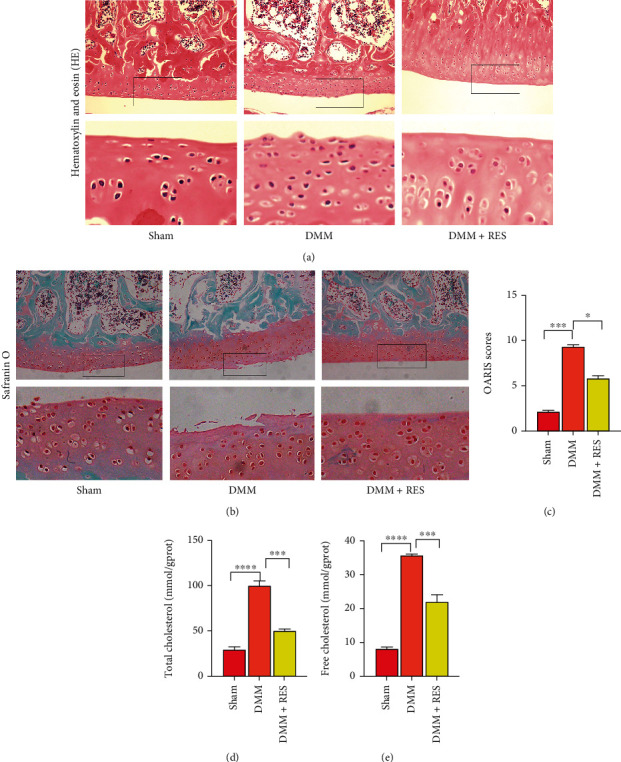
RES ameliorates OA development in DMM mice models. (a, b) Hematoxylin and eosin and safranin-O staining of different groups (scale bar: 300 *μ*m). (c) OA score for cartilage. (d, e) The respective cholesterol assay kits quantified cartilage tissues' free and total cholesterol levels. The values represent the mean ± standard deviation of three independent experiments. ^∗^*p* < 0.05, ^∗∗^*p* < 0.01, ^∗∗∗^*p* < 0.001, and ^∗∗∗∗^*p* < 0.0001.

According to the H&E and SO staining experiments results, cartilage in the DMM group showed significant erosion, proteoglycan loss, and chondrocyte hypertrophy (Figures 6(a) and 6(b)). The cartilage in the RES group had a smoother surface and experienced less proteoglycan loss than the DMM group (Figures 6(a) and 6(b)). Additionally, the DMM experimental groups' OARSI score decreased significantly after RES treatment (Figure 6(c)). This was consistent with the results of H&E and SO staining.

**Figure 7:**
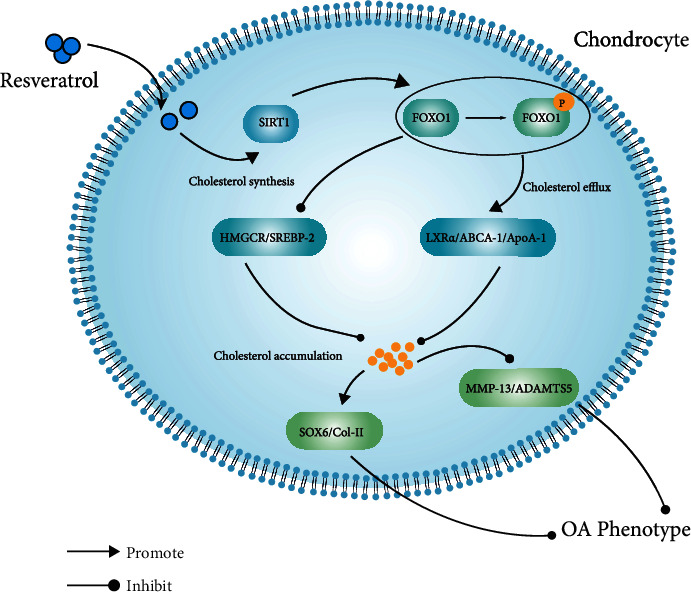
RES reverses cartilage OA phenotype by improving cholesterol accumulation in articular cartilage of DMM mice Figure 7. Resveratrol attenuates the degeneration of osteoarthritic chondrocytes by regulating the SIRT1/FoxO1-mediated cholesterol metabolism in osteoarthritic chondrocytes.

Additionally, the cholesterol content in chondrocytes was determined, and the results showed that the cholesterol content of cartilage in the RES group was significantly lower than that in the DMM group (Figures 7(k) and 7(l)). The results showed that RES reduced the accumulation of cholesterol in the cartilage of OA rats via the SIRT1/FoxO1 pathway, thereby alleviating the progression of OA.

## 4. Discussion

The prevalence of OA is increasing every year, and it affects a significant portion of the elderly population globally [19]. Serum cholesterol levels are associated with OA in humans [20–22]. However, the role of cholesterol metabolism in the development of OA is unclear. Chadha found that the pathogenesis of OA was not merely a localized lesion of the articular cartilage but a systemic metabolic abnormality [23]. Furthermore, Tabas reported that cellular cholesterol buildup affects the function and structure of cell membranes and can even cause cell lysis [24]. Thus, dysregulation of cholesterol metabolism is critical to the development of OA.

In the present study, an in-depth investigation of the clinical specimens of OA showed that *SIRT1* and *p-FoxO1* were downregulated in OA chondrocytes and caused cholesterol buildup in OA chondrocytes, indicating that the SIRT1-FoxO1 pathway is involved in cholesterol buildup in chondrocytes. Notably, RES reduced IL-1*β*-induced cholesterol accumulation and OA phenotype in chondrocytes by activating the SIRT1-FoxO1 pathway. Therefore, RES can reverse cholesterol accumulation in OA chondrocytes by activating the SIRT1-FoxO1 pathway, thereby improving the OA degradation.

RES is a strong agonist of *SIRT1*, and RES has a positive effect on OA [25]. *SIRT1* regulates *FoxO1* expression in the skeletal muscle and adipocytes [26–28], and FoxO1 is highly expressed in cartilage. We report SIRT1-FoxO1 to be closely associated with the progression of OA, given that its expression was significantly reduced in the cartilage tissue of patients with OA. Furthermore, RES promoted IL-1*β*-induced *COL-II* and *SOX6* in chondrocytes and inhibited the expression of *MMP13* and *ADAMTS5*. *COL-II* and *SOX6* are the major genes involved in cartilage extracellular matrix synthesis, and *MMP13* and *ADAMTS5* are the major genes involved in ECM decomposition [29]. RES can maintain chondrocyte matrix synthesis and inhibit matrix degradation via the SIRT1-FoxO1 pathway, thereby alleviating the OA phenotype in IL-1*β*-induced chondrocytes. Meanwhile, the *in vivo* study results showed that RES ameliorates OA development in DMM mice models.

With the significant scientific progress achieved in recent years, the potential therapeutic effects of RES on OA have gradually been uncovered. However, the effect of RES on cholesterol accumulation in OA chondrocytes has rarely been studied. The balance of cellular cholesterol metabolism is crucial to the stability of chondrocyte function [12, 30]. RES can minimize cholesterol accumulation in vascular endothelial cells, thereby improving atherosclerosis [31]. Sun et al. found that *SIRT1* inhibits the expression of the hepatocyte cholesterol synthesis genes *SREBP-2* and *HMGCR* by promoting the phosphorylation of *FoxO1* [15]. Emerging evidence suggests that the SIRT1/FoxO1 pathway can regulate cellular lipid metabolism. Given the role of RES on cholesterol metabolism and the fact that it is also an agonist of SIRT1, we hypothesized that RES may regulate cholesterol metabolism in chondrocytes by activating the SIRT1/FoxO1 pathway.

As the accumulation of intracellular cholesterol causes cellular dysfunction, most cells have a strictly regulated cholesterol metabolism system to avoid increases in intracellular cholesterol levels [32]. Tsezou et al. found a significant decrease in the expression levels of the cholesterol efflux genes in OA chondrocytes (*LXRα*, *ABCA1*, and *ApoA1*) [6], corroborating our results. The expression levels of cholesterol synthesis genes (*SREBP-2* and *HMGCR*) in OA chondrocytes increased, implicating the disruption of cholesterol metabolism as an important factor in the development of OA. To explore the protective mechanisms of RES in chondrocytes, we investigated the role of RES in gene expression related to cholesterol synthesis and efflux. With the addition of RES, cholesterol efflux was promoted via the activation of the SIRT1-FoxO1 signaling pathway, and cholesterol synthesis was inhibited. Moreover, the addition of inhibitors EX-527 and AS enhanced cholesterol synthesis and attenuated cholesterol efflux considerably. RES could regulate the expression of chondrocyte cholesterol synthesis and efflux-related genes and ameliorate cholesterol accumulation in IL-1*β*-induced chondrocytes. Additionally, we investigated the effects of RES on total and free cholesterol levels in chondrocytes to verify the above results. The effect of the SIRT1-FoxO1 pathway on cholesterol metabolism has been similarly studied in other cells. Recent studies have shown that downregulation of cholesterol synthesis by activation of SIRT1/FoxO1/SREBP-2 attenuates nonalcoholic hepatic steatosis [33]. Another study showed that promoting SIRT1/FoxO1/ATG14-dependent autophagy prevents dysfunction of lipid metabolism in HepG2 injury [34]. Thus, RES alleviated IL-1*β*-induced cholesterol accumulation in chondrocytes via the SIRT1-FoxO1 pathway.

DMM is a commonly used *in vivo* OA model. The DMM group showed articular cartilage erosion, cell hypertrophy, massive proteoglycan loss, and cholesterol buildup in our study. Nonetheless, all these phenomena significantly improved after RES treatment. These *in vivo* and *in vitro* findings suggest that RES can reduce cartilage cholesterol accumulation in OA cartilage through the SIRT1/foxO1 pathway, thereby alleviating the progression of OA (Figure 7).

## 5. Conclusion

The results of this study suggest that RES improves IL-1*β*-induced chondrocyte cholesterol buildup in chondrocytes by activating the SIRT1-FoxO1 pathway, which attenuates the IL-1*β*-induced OA phenotype. Furthermore, our *in vivo* results suggest that RES attenuates cholesterol buildup in the cartilage of the DMM model, thereby delaying the progression of OA. These results provide a theoretical basis for RES treatment of OA.

## Figures and Tables

**Table 1:** Primer sequences.

Gene	Forward primer (5′-3′)	Reverse primer (5′-3′)
Rat GAPDH	5′-ACAGCAACAGGGTGGTGGAC-3′	5′-TTTGAGGGTGCAGCGAACTT-3′
Rat COL-II	5′-ATGTTACCGTCAGCACCACC-3′	5′-GACAAGGGCTTGAGAGGCAC-3′
Rat MMP13	5′-CCCGAGACCTCATGTTCATCT-3′	5′-CTTCTTCTATGAGGCGGGGAT-3′
Rat Foxo1	5′-CCATGCCTCACACATCTGCC-3′	5′TTAAAATCCAAGGTATCTCCGTCCA-3′
Rat APOA1	5′-AACGCGAAGGAGATGCAAAG-3′	5′-AGGGTGGTTCTTGATCTCGG-3′
Rat LXR*α*	5′-GTGCCTGATGTTTCTCCTGAC-3′	5′-ATACACTGCATAGCTCGTTCC-3′
Rat ABCA1	5′-GCAGCGACCATGAAAGTGAC-3′	5′-GAGGCGGTCATCAATCTCGT-3′
Rat SREBP-2	5′-CGAACTGGGCGATGGATGAGA-3′	5′-TCTCCCACTTGATTGCTGACA-3′
Rat HMGCR	5′-CCTCCATTGAGATCCGGAGG-3′	5′-ACCGGGTTATCGTGAGGATG-3′
Rat SIRT1	5′-TGCCATCATGAAGCCAGAGA-3′	5′-CATCGCAGTCTCCAAGAAGC-3′
Rat ADAMTS5	5′-CACCAAAGCCAGCACATAGG-3′	5′-TTTAACTCAAGCTGCCTCGC-3′
Rat SOX6	5′-CTTCCCAGATCGCCTACACC-3′	5′-CTGCGTGGCCATAATAGGGT-3′
Homo GAPDH	5′-TCAAGAAGGTGGTGAAGCAGG-3′	5′-TCAAAGGTGGAGGAGTGGGT-3′
Homo SIRT1	5′-AGCAGATTAGTAGGCGGCTT-3′	5′-GACTCTGGCATGTCCCACTA-3′
Homo ABCA1	5′-TGCCTCCTCCACAAAGAAAACAAAA-3′	5′-CCGCCATACCTAAACTCATTCAC-3′
Homo APOA1	5′-GACAGCGTGACCTCCACCTT-3′	5′-ATCTCCTCCTGCCACTTCTTC-3′
Homo LXR*α*	5′-CCTCGGGCTTCCACTACAATGTTC-3′	5′-TCCTCTTGCCGCTTCAGTTTCTTCA-3′
Homo SREBP2	5′-GGTGAAACCTCAGGCCAAGAAGAAG-3′	5′-CCCCACAGAGTCCACAAAAGAA-3′
Homo HMGCR	5′-GTTTCCCTCTGGCAGTTTTATCTCT-3′	5′-TCTTTCTCGGTTTATCCCTGTCTCT-3′
Homo FOXO1	5′-TGAAACGAGCAACTATCAAAGAC-3′	5′-ATAAAAGAACCAGATGGAGGACT-3′

**Table 2:** Antibodies used in the experiments.

Antibodies	Company	Catalog	Dilution ratio
COL-II (142KD)	Affinity	AF0135	1 : 1500
ADAMTS5 (72KD)	Affinity	DF7470	1 : 1000
SREBP-2 (124KD)	Affinity	DF7601	1 : 2000
HMGCR (97KD)	Abcam	Ab174830	1 : 4000
SIRT1 (120KD)	ProteinTech Group	60303-1-Ig	1 : 5000
FoxO1 (74KD)	ABclonal	A2934	1 : 1000
p-FoxO1 (78KD)	Affinity	AF3416	1 : 1000
SOX6 (49KD)	Abcam	ab195966	1 : 1000
MMP-13 (54KD)	Abcam	Ab39012	1 : 3000
GAPDH (37KD)	GOODHERE	AB-P-R 001	1 : 1000
ABCA1 (254KD)	Abcam	Ab66217	1 : 1000
APOA1 (31KD)	Affinity	DF6264	1 : 1000
LXR*α* (50KD)	Affinity	DF6864	1 : 1000

## Data Availability

The data used to support the findings of this study are included within the article.
